# Crystal structure of fluroxypyr

**DOI:** 10.1107/S2056989016018533

**Published:** 2016-11-25

**Authors:** Hyunjin Park, Myong Yong Choi, Eunjin Kwon, Tae Ho Kim

**Affiliations:** a Department of Chemistry (BK21 plus) and Research Institute of Natural Sciences, Gyeongsang National University, Jinju 52828, Republic of Korea

**Keywords:** crystal structure, fluroxypyr, herbicide, pyridine, hydrogen bonds

## Abstract

In the title pyridine herbicide {systematic name: 2-[(4-amino-3,5-di­chloro-6-fluoro­pyridin-2-yl)­oxy]acetic acid}, C_7_H_5_Cl_2_FN_2_O_3_, the mean plane of the carb­oxy­lic acid substituent and the pyridyl ring plane subtend a dihedral angle of 77.5 (1)°. In the crystal, pairs of O—H⋯O hydrogen bonds form inversion dimers with *R*_2_^2^(8) ring motifs. These are extended into chains along [011] by N—H⋯F hydrogen bonds. In addition, inter­molecular N—H⋯O hydrogen bonds and weak π–π inter­actions [ring centroid separation = 3.4602 (9) Å] connect these chains into a three-dimensional network.

## Abstract 2

The title compound {systematic name: 2-[(4-amino-3,5-di­chloro-6-fluoro­pyridin-2-yl)­oxy]acetic acid}, C_7_H_5_Cl_2_FN_2_O_3_, is a pyridine herbicide. In the crystal, N—H⋯O, O—H⋯O and N—H⋯F hydrogen bonds and weak π–π inter­actions connect chains of mol­ecules into a three-dimensional network.

## Chemical context   

Fluroxypyr belongs to the pyridine family of herbicides. It is widely used on cereal crops, olive trees and fallow croplands to control broad-leaf weeds (Moreno-Castilla *et al.*, 2012 ▸; Wang *et al.*, 2011 ▸). Pyridine herbicides such as fluroxypyr are effective and popular chemicals for post-emergence broad-leaf weed control, particularly in turf during cool seasons. The efficacy of this herbicide may be affected by environmental conditions including the relative humidity, temperature and soil moisture. Because of this, its application often provides inconsistent broad-leaf weed control in winter or early spring (Reed & McCullough, 2012 ▸). Until now, its crystal structure had not been reported and we describe it herein.
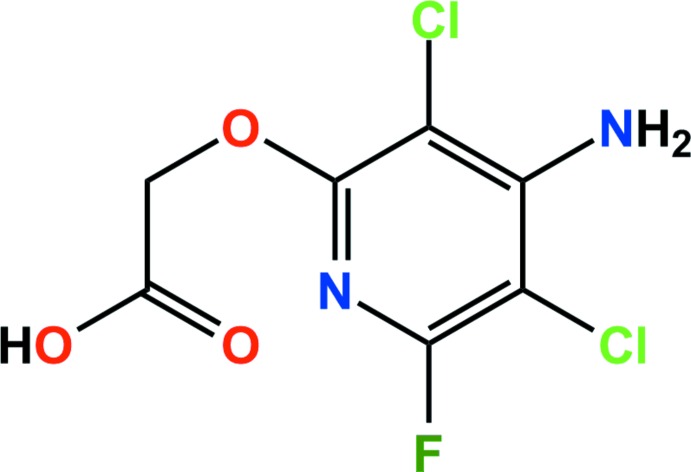


## Structural commentary   

**Figure 1:**
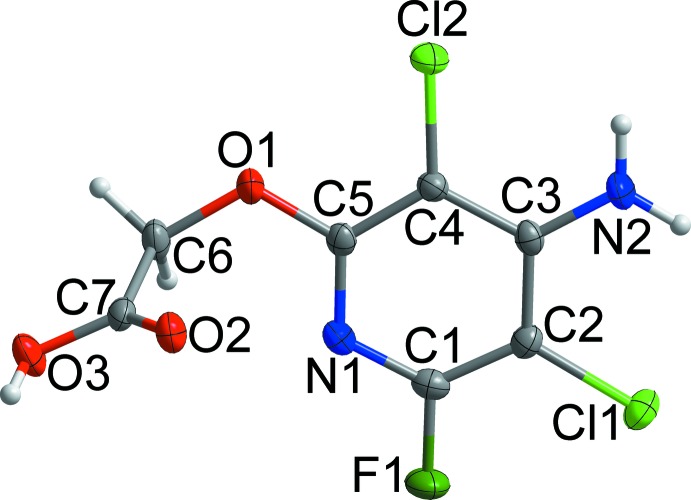
The structure of the title compound, with displacement ellipsoids drawn at the 50% probability level. H atoms are shown as small spheres of arbitrary radius.

The structure of fluroxypyr is shown in Fig. 1 ▸. The dihedral angle between the mean plane of the carb­oxy­lic acid group (C6/C7/O2/O3) and the pyridyl ring (N1/C1–C5) is 77.5 (1)°. All bond lengths and bond angles are normal and comparable to those observed in the crystal structure of a related pyridine-containing herbicide (Cho *et al.*, 2015 ▸).

## Supra­molecular features   

**Figure 2:**
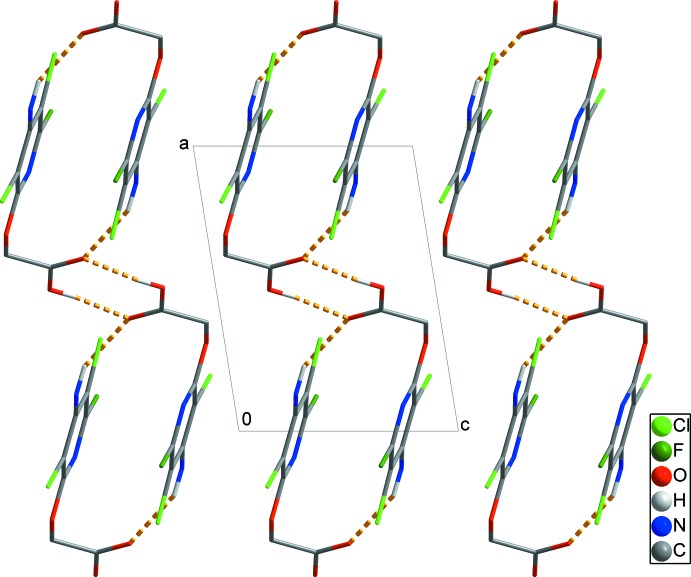
A view along the *b* axis of the crystal packing of the title compound. The chains are formed through inter­molecular O—H⋯O and N—H⋯O hydrogen bonds (yellow dashed lines). H atoms not involved in these inter­actions have been omitted for clarity.

**Figure 3:**
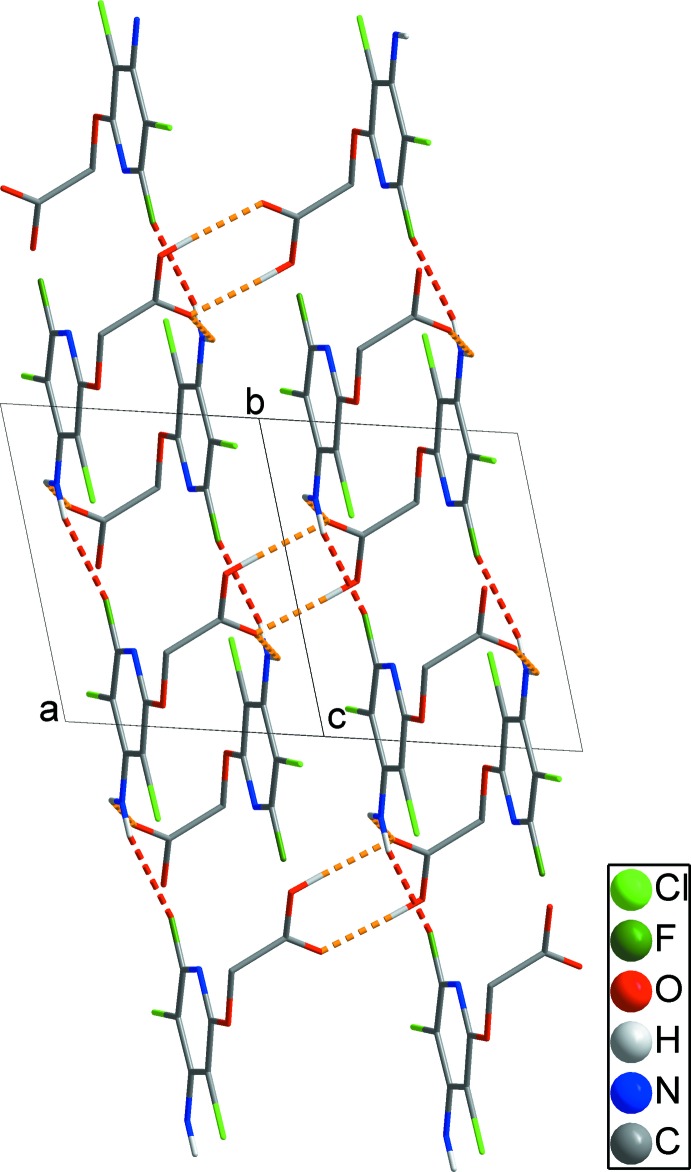
The two-dimensional network formed through inter­molecular N—H⋯F hydrogen bonds (red dashed lines). Inter­molecular O/N–H⋯O hydrogen bonds within a chain are shown as yellow dashed lines. H atoms not involved in these inter­actions have been omitted for clarity.

**Figure 4:**
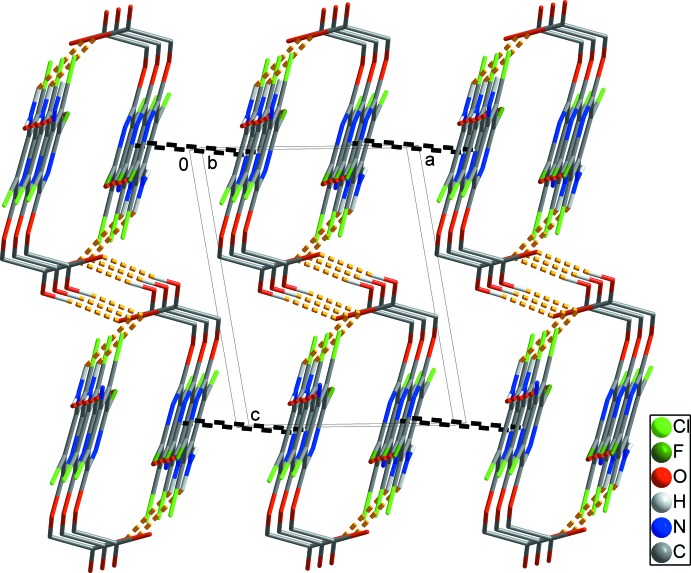
A packing diagram showing the three-dimensional architecture formed by weak π–π inter­actions (black dashed lines). Inter­molecular O—H⋯O, N—H⋯O and N—H⋯F hydrogen bonds within a sheet are shown as yellow and red dashed lines. H atoms not involved in inter­molecular inter­actions have been omitted for clarity.

In the crystal, the solid-state structure is stabilized by pairs of N2—H2*B*⋯O2 hydrogen bonds, forming inversion dimers with 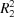
(18) ring motifs (Table 1 ▸ and Fig. 2 ▸). These dimers are linked by pairs of O3—H3⋯O2^i^/O2 hydrogen bonds that form classical carb­oxy­lic-acid-based inversion dimers with 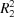
(8) ring motifs. These contacts form chains propagating along [011] (yellow dashed lines in Fig. 2 ▸). In addition, inter­molecular N2—H2*A*⋯F1 hydrogen bonds connect these chains, yielding sheets extending parallel to the *bc* plane (red dashed line in Fig. 3 ▸). These sheets are further linked by weak inter­molecular π–π inter­actions between the pyridyl rings (N1/C1–C5) [*Cg*1⋯*Cg*1^iv^ = 3.4602 (9) Å; symmetry code: (iv) −*x*, −*y* + 2, −*z*], resulting in a three-dimensional network structure (black dashed lines in Fig. 4 ▸).

## Database survey   

We have reported the crystal structure of several pesticides including compounds with pyridine rings (Cho *et al.*, 2015 ▸; Kang *et al.*, 2015 ▸; Kwon *et al.*, 2016 ▸; Park *et al.*, 2016 ▸). In addition, a database search (CSD; Groom *et al.*, 2006 ▸) yielded two other comparable structures, 2-[(3,5,6-tri­chloro­pyridin-2-yl)­oxy]acetic acid (Cho *et al.*, 2014 ▸) and 2,4,5-tri­chloro­phen­oxy­acetic acid (Smith *et al.*, 1976 ▸).

## Synthesis and crystallization   

The title compound was purchased from Dr. Ehrenstorfer GmbH. Colorless single crystals suitable for X-ray diffraction were obtained from a CH_3_CN solution by slow evaporation at room temperature.

## Refinement   

Crystal data, data collection and structure refinement details are summarized in Table 2 ▸. All H atoms were positioned geometrically and refined using a riding model with *d*(O—H) = 0.84 Å, *U*_iso_ = 1.5*U*_eq_(C) for the O—H group, *d*(N—H) = 0.88 Å, *U*_iso_ = 1.2*U*_eq_(C) for the amine group, and *d*(C—H) = 0.99 Å, *U*_iso_ = 1.2*U*_eq_(C) for the CH_2_ group.

## Supplementary Material

Crystal structure: contains datablock(s) I, New_Global_Publ_Block. DOI: 10.1107/S2056989016018533/sj5515sup1.cif

Structure factors: contains datablock(s) I. DOI: 10.1107/S2056989016018533/sj5515Isup2.hkl

Supporting information file. DOI: 10.1107/S2056989016018533/sj5515Isup3.cml

CCDC reference: 1518035

Additional supporting information: crystallographic information; 3D view; checkCIF report

## Figures and Tables

**Table 1:** Hydrogen-bond geometry (Å, °)

*D*—H⋯*A*	*D*—H	H⋯*A*	*D*⋯*A*	*D*—H⋯*A*
O3—H3⋯O2^i^	0.84	1.84	2.6801 (15)	174
N2—H2*A*⋯F1^ii^	0.88	2.39	2.9950 (15)	126
N2—H2*B*⋯O2^iii^	0.88	2.25	3.0201 (16)	146

Symmetry codes: (i) 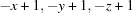; (ii) 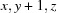; (iii) 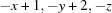.

**Table 2:** Experimental details

Crystal data	
Chemical formula	C_7_H_5_Cl_2_FN_2_O_3_
*M*_r_	255.03
Crystal system, space group	Triclinic, *P* 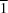
Temperature (K)	173
*a*, *b*, *c* (Å)	7.1116 (9), 7.6131 (9), 8.9414 (11)
α, β, γ (°)	86.927 (6), 80.354 (6), 72.587 (5)
*V* (Å^3^)	455.38 (10)
*Z*	2
Radiation type	Mo *K*α
μ (mm^−1^)	0.72
Crystal size (mm)	0.23 × 0.22 × 0.04

Data collection	
Diffractometer	Bruker APEXII CCD
Absorption correction	Multi-scan (*SADABS*; Bruker, 2014 ▸)
*T*_min_, *T*_max_	0.690, 0.746
No. of measured, independent and observed [*I* > 2σ(*I*)] reflections	8052, 2092, 1972
*R*_int_	0.023
(sin θ/λ)_max_ (Å^−1^)	0.650

Refinement	
*R*[*F* ^2^ > 2σ(*F* ^2^)], *wR*(*F* ^2^), *S*	0.028, 0.076, 1.12
No. of reflections	2092
No. of parameters	137
H-atom treatment	H-atom parameters constrained
Δρ_max_, Δρ_min_ (e Å^−3^)	0.29, −0.38

Computer programs: *APEX2* and *SAINT* (Bruker, 2014 ▸), *SHELXS97* and *SHELXTL* (Sheldrick, 2008 ▸), *SHELXL2014* (Sheldrick, 2015 ▸) and *DIAMOND* (Brandenburg, 2010 ▸).

## supplementary crystallographic information

### Crystal data

**Table:**

C_7_H_5_Cl_2_FN_2_O_3_	*Z* = 2
*M**_r_* = 255.03	*F*(000) = 256
Triclinic, *P*1	*D*_x_ = 1.860 Mg m^−^^3^
*a* = 7.1116 (9) Å	Mo *K*α radiation, λ = 0.71073 Å
*b* = 7.6131 (9) Å	Cell parameters from 6070 reflections
*c* = 8.9414 (11) Å	θ = 2.8–27.5°
α = 86.927 (6)°	µ = 0.72 mm^−^^1^
β = 80.354 (6)°	*T* = 173 K
γ = 72.587 (5)°	Plate, colourless
*V* = 455.38 (10) Å^3^	0.23 × 0.22 × 0.04 mm

### Data collection

**Table:**

Bruker APEXII CCD diffractometer	1972 reflections with *I* > 2σ(*I*)
φ and ω scans	*R*_int_ = 0.023
Absorption correction: multi-scan (SADABS; Bruker, 2014)	θ_max_ = 27.5°, θ_min_ = 2.3°
*T*_min_ = 0.690, *T*_max_ = 0.746	*h* = −9→8
8052 measured reflections	*k* = −9→9
2092 independent reflections	*l* = −10→11

### Refinement

**Table:**

Refinement on *F*^2^	0 restraints
Least-squares matrix: full	Hydrogen site location: inferred from neighbouring sites
*R*[*F*^2^ > 2σ(*F*^2^)] = 0.028	H-atom parameters constrained
*wR*(*F*^2^) = 0.076	*w* = 1/[σ^2^(*F*_o_^2^) + (0.0371*P*)^2^ + 0.2062*P*] where *P* = (*F*_o_^2^ + 2*F*_c_^2^)/3
*S* = 1.12	(Δ/σ)_max_ < 0.001
2092 reflections	Δρ_max_ = 0.29 e Å^−^^3^
137 parameters	Δρ_min_ = −0.38 e Å^−^^3^

### Special details

**Table:**

Geometry. All esds (except the esd in the dihedral angle between two l.s. planes) are estimated using the full covariance matrix. The cell esds are taken into account individually in the estimation of esds in distances, angles and torsion angles; correlations between esds in cell parameters are only used when they are defined by crystal symmetry. An approximate (isotropic) treatment of cell esds is used for estimating esds involving l.s. planes.

### Fractional atomic coordinates and isotropic or equivalent isotropic displacement parameters (Å^2^)

**Table:**

	*x*	*y*	*z*	*U*_iso_*/*U*_eq_	
Cl1	0.43701 (6)	0.90760 (5)	−0.32138 (4)	0.02990 (12)	
Cl2	0.09018 (6)	1.29195 (5)	0.19394 (4)	0.02692 (11)	
F1	0.37852 (14)	0.60390 (12)	−0.12163 (11)	0.0298 (2)	
O1	0.08333 (16)	0.93076 (14)	0.31061 (11)	0.0234 (2)	
O2	0.41952 (15)	0.68493 (14)	0.39455 (11)	0.0225 (2)	
O3	0.24193 (16)	0.50096 (15)	0.50373 (13)	0.0278 (2)	
H3	0.3515	0.4408	0.5295	0.042*	
N1	0.23296 (18)	0.76320 (16)	0.09169 (14)	0.0202 (2)	
N2	0.2865 (2)	1.24580 (17)	−0.12976 (14)	0.0244 (3)	
H2A	0.2414	1.3515	−0.0813	0.029*	
H2B	0.3429	1.2417	−0.2255	0.029*	
C1	0.3133 (2)	0.76709 (19)	−0.05118 (17)	0.0202 (3)	
C2	0.3356 (2)	0.91926 (19)	−0.13244 (15)	0.0192 (3)	
C3	0.26931 (19)	1.08983 (18)	−0.05718 (15)	0.0177 (3)	
C4	0.1811 (2)	1.08826 (18)	0.09480 (15)	0.0176 (3)	
C5	0.16793 (19)	0.92352 (19)	0.16331 (15)	0.0178 (3)	
C6	0.0683 (2)	0.7624 (2)	0.38160 (17)	0.0242 (3)	
H6A	0.0248	0.6917	0.3114	0.029*	
H6B	−0.0345	0.7900	0.4736	0.029*	
C7	0.2634 (2)	0.64680 (19)	0.42518 (15)	0.0197 (3)	

### Atomic displacement parameters (Å^2^)

**Table:**

	*U*^11^	*U*^22^	*U*^33^	*U*^12^	*U*^13^	*U*^23^
Cl1	0.0345 (2)	0.0351 (2)	0.01671 (18)	−0.00767 (16)	0.00124 (14)	−0.00224 (14)
Cl2	0.0359 (2)	0.01784 (17)	0.02395 (19)	−0.00397 (14)	−0.00232 (14)	−0.00355 (13)
F1	0.0372 (5)	0.0182 (4)	0.0318 (5)	−0.0048 (4)	−0.0035 (4)	−0.0076 (4)
O1	0.0270 (5)	0.0214 (5)	0.0177 (5)	−0.0044 (4)	0.0008 (4)	0.0058 (4)
O2	0.0245 (5)	0.0241 (5)	0.0208 (5)	−0.0101 (4)	−0.0048 (4)	0.0060 (4)
O3	0.0243 (5)	0.0232 (5)	0.0355 (6)	−0.0081 (4)	−0.0051 (5)	0.0130 (4)
N1	0.0209 (6)	0.0169 (5)	0.0232 (6)	−0.0058 (4)	−0.0055 (5)	0.0035 (4)
N2	0.0318 (7)	0.0200 (6)	0.0216 (6)	−0.0103 (5)	−0.0013 (5)	0.0055 (5)
C1	0.0195 (6)	0.0167 (6)	0.0242 (7)	−0.0035 (5)	−0.0058 (5)	−0.0025 (5)
C2	0.0190 (6)	0.0226 (7)	0.0158 (6)	−0.0060 (5)	−0.0025 (5)	−0.0002 (5)
C3	0.0170 (6)	0.0189 (6)	0.0183 (6)	−0.0065 (5)	−0.0053 (5)	0.0037 (5)
C4	0.0186 (6)	0.0155 (6)	0.0181 (6)	−0.0038 (5)	−0.0035 (5)	0.0002 (5)
C5	0.0150 (6)	0.0200 (6)	0.0173 (6)	−0.0036 (5)	−0.0037 (5)	0.0033 (5)
C6	0.0236 (7)	0.0258 (7)	0.0218 (7)	−0.0081 (6)	−0.0014 (5)	0.0090 (6)
C7	0.0252 (7)	0.0202 (6)	0.0132 (6)	−0.0073 (5)	−0.0005 (5)	0.0007 (5)

### Geometric parameters (Å, º)

**Table:**

Cl1—C2	1.7181 (14)	N2—C3	1.3506 (17)
Cl2—C4	1.7216 (14)	N2—H2A	0.8800
F1—C1	1.3403 (16)	N2—H2B	0.8800
O1—C5	1.3499 (17)	C1—C2	1.370 (2)
O1—C6	1.4243 (17)	C2—C3	1.4080 (19)
O2—C7	1.2143 (17)	C3—C4	1.3990 (19)
O3—C7	1.3158 (17)	C4—C5	1.3877 (19)
O3—H3	0.8400	C6—C7	1.505 (2)
N1—C1	1.3117 (19)	C6—H6A	0.9900
N1—C5	1.3268 (18)	C6—H6B	0.9900
			
C5—O1—C6	117.32 (11)	C5—C4—C3	119.78 (12)
C7—O3—H3	109.5	C5—C4—Cl2	121.01 (11)
C1—N1—C5	116.03 (12)	C3—C4—Cl2	119.20 (10)
C3—N2—H2A	120.0	N1—C5—O1	119.54 (12)
C3—N2—H2B	120.0	N1—C5—C4	123.51 (13)
H2A—N2—H2B	120.0	O1—C5—C4	116.96 (12)
N1—C1—F1	115.22 (12)	O1—C6—C7	112.12 (12)
N1—C1—C2	126.49 (13)	O1—C6—H6A	109.2
F1—C1—C2	118.29 (13)	C7—C6—H6A	109.2
C1—C2—C3	118.00 (13)	O1—C6—H6B	109.2
C1—C2—Cl1	122.11 (11)	C7—C6—H6B	109.2
C3—C2—Cl1	119.88 (10)	H6A—C6—H6B	107.9
N2—C3—C4	122.43 (12)	O2—C7—O3	124.35 (13)
N2—C3—C2	121.39 (12)	O2—C7—C6	124.46 (13)
C4—C3—C2	116.17 (12)	O3—C7—C6	111.18 (12)
			
C5—N1—C1—F1	179.85 (11)	C2—C3—C4—Cl2	−178.35 (10)
C5—N1—C1—C2	−0.2 (2)	C1—N1—C5—O1	179.97 (12)
N1—C1—C2—C3	0.9 (2)	C1—N1—C5—C4	0.1 (2)
F1—C1—C2—C3	−179.10 (12)	C6—O1—C5—N1	−0.13 (18)
N1—C1—C2—Cl1	−178.05 (11)	C6—O1—C5—C4	179.71 (12)
F1—C1—C2—Cl1	1.94 (19)	C3—C4—C5—N1	−0.9 (2)
C1—C2—C3—N2	179.92 (13)	Cl2—C4—C5—N1	179.01 (10)
Cl1—C2—C3—N2	−1.09 (18)	C3—C4—C5—O1	179.27 (11)
C1—C2—C3—C4	−1.55 (19)	Cl2—C4—C5—O1	−0.82 (17)
Cl1—C2—C3—C4	177.43 (10)	C5—O1—C6—C7	78.48 (15)
N2—C3—C4—C5	−179.93 (12)	O1—C6—C7—O2	−4.9 (2)
C2—C3—C4—C5	1.56 (19)	O1—C6—C7—O3	173.75 (12)
N2—C3—C4—Cl2	0.16 (18)		

### Hydrogen-bond geometry (Å, º)

**Table:**

*D*—H···*A*	*D*—H	H···*A*	*D*···*A*	*D*—H···*A*
O3—H3···O2^i^	0.84	1.84	2.6801 (15)	174
N2—H2*A*···F1^ii^	0.88	2.39	2.9950 (15)	126
N2—H2*B*···O2^iii^	0.88	2.25	3.0201 (16)	146

Symmetry codes: (i) −*x*+1, −*y*+1, −*z*+1; (ii) *x*, *y*+1, *z*; (iii) −*x*+1, −*y*+2, −*z*.

## References

[bb1] Brandenburg, K. (2010). *DIAMOND*. Crystal Impact GbR, Bonn, Germany.

[bb2] Bruker (2014). *APEX2*, *SAINT* and *SADABS*. Bruker AXS Inc., Madison, Wisconsin, USA.

[bb3] Cho, S., Kim, J., Jeon, Y. & Kim, T. H. (2014). *Acta Cryst.* E**70**, o940. 10.1107/S160053681401681X PMC4186132 25309266

[bb4] Cho, S., Kim, J., Lee, S. & Kim, T. H. (2015). *Acta Cryst.* E**71**, o55. 10.1107/S2056989014026632 PMC4331875 25705506

[bb14] Groom, C. R., Bruno, I. J., Lightfoot, M. P. & Ward, S. C. (2016). *Acta Cryst* B**72**, 171–179. 10.1107/S2052520616003954 PMC4822653 27048719

[bb5] Kang, G., Kim, J., Park, H. & Kim, T. H. (2015). *Acta Cryst.* E**71**, o588. 10.1107/S2056989015013481 PMC4571411 26396811

[bb6] Kwon, E., Kim, J., Park, H. & Kim, T. H. (2016). *Acta Cryst.* E**72**, 1468–1470. 10.1107/S2056989016014845 PMC5050778 27746943

[bb7] Moreno-Castilla, C., López-Ramón, M. V., Pastrana-Martínez, L. M., Álvarez-Merino, M. A. & Fontecha-Cámara, M. A. (2012). *Adsorption*, **18**, 173–179.

[bb8] Park, H., Kwon, E., Yoon, I. & Kim, J. (2016). *Acta Cryst.* E**72**, 1610–1613. 10.1107/S2056989016016662 PMC5095845 27840720

[bb9] Reed, T. V. & McCullough, P. E. (2012). *Hort. Sci.* **47**, 1548–1549.

[bb10] Sheldrick, G. M. (2008). *Acta Cryst.* A**64**, 112–122. 10.1107/S0108767307043930 18156677

[bb11] Sheldrick, G. M. (2015). *Acta Cryst.* C**71**, 3–8.

[bb12] Smith, G., Kennard, C. H. L. & White, A. H. (1976). *Aust. J. Chem.* **29**, 2727–2730.

[bb13] Wang, L., Xu, J., Zhao, P. & Pan, C. (2011). *Bull. Environ. Contam. Toxicol.* **86**, 449–453. 10.1007/s00128-011-0218-y 21340457

